# Bright-field holography: cross-modality deep learning enables snapshot 3D imaging with bright-field contrast using a single hologram

**DOI:** 10.1038/s41377-019-0139-9

**Published:** 2019-03-06

**Authors:** Yichen Wu, Yilin Luo, Gunvant Chaudhari, Yair Rivenson, Ayfer Calis, Kevin de Haan, Aydogan Ozcan

**Affiliations:** 1 Electrical and Computer Engineering Department, University of California, Los Angeles, CA 90095 USA; 2 Bioengineering Department, University of California, Los Angeles, CA 90095 USA; 3 California Nano Systems Institute (CNSI), University of California, Los Angeles, CA 90095 USA; 4 David Geffen School of Medicine, University of California, Los Angeles, CA 90095 USA

**Keywords:** Imaging and sensing, Microscopy, Biophotonics

## Abstract

Digital holographic microscopy enables the 3D reconstruction of volumetric samples from a single-snapshot hologram. However, unlike a conventional bright-field microscopy image, the quality of holographic reconstructions is compromised by interference fringes as a result of twin images and out-of-plane objects. Here, we demonstrate that cross-modality deep learning using a generative adversarial network (GAN) can endow holographic images of a sample volume with bright-field microscopy contrast, combining the volumetric imaging capability of holography with the speckle- and artifact-free image contrast of incoherent bright-field microscopy. We illustrate the performance of this “bright-field holography” method through the snapshot imaging of bioaerosols distributed in 3D, matching the artifact-free image contrast and axial sectioning performance of a high-NA bright-field microscope. This data-driven deep-learning-based imaging method bridges the contrast gap between coherent and incoherent imaging, and enables the snapshot 3D imaging of objects with bright-field contrast from a single hologram, benefiting from the wave-propagation framework of holography.

Digital holographic microscopy encodes the volumetric information of a sample into a single 2D diffraction pattern. Thus, digital holographic microscopy enables the reconstruction of volumetric samples from a single-hologram measurement without any mechanical scanning^1–6^. However, for most practical applications, holographic images cannot match the speckle- and artifact-free image contrast of incoherent bright-field microscopy. Some of these holographic artifacts include twin-image and self-interference artifacts, which are related to missing phase information; additional artifacts appear due to the long coherence length/diameter of the illumination source, which creates speckle and background interference from out-of-focus or unwanted objects in the optical path. Stated differently, since the point-spread function (PSF) of a coherent imaging system has nondiminishing ripples along both the lateral and axial directions, out-of-focus objects will create interference fringes overlapping with the in-focus objects in the holographic reconstruction, which degrades the image contrast when reconstructing volumetric samples. These issues can be partially mitigated by using different holographic reconstruction methods and sometimes also by using additional measurements^4,7–14^.

Here, we use a deep neural network to perform cross-modality image transformation from a digitally backpropagated hologram corresponding to a given depth within the sample volume into an image that is equivalent to a bright-field microscopy image acquired at the same depth (Fig. 1). Since a single hologram is used to digitally propagate image information to different sections of the sample to virtually generate a bright-field equivalent image of each section, this approach combines the snapshot volumetric imaging capability of digital holography with the speckle- and artifact-free image contrast and axial sectioning performance of bright-field microscopy. Following its training, the deep neural network has learned the statistical image transformation between a holographic imaging system and an incoherent bright-field microscope; therefore, we refer to this approach as “Bright-field Holography”. In some sense, deep learning brings together the best of both worlds by fusing the advantages of both the holographic and incoherent bright-field imaging modalities.

**Fig. 1:**
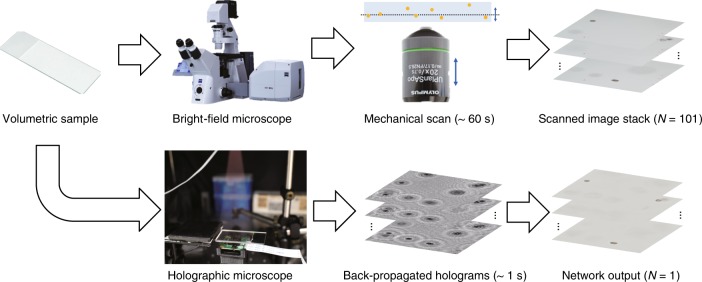
Bright-field holography. High-contrast bright-field imaging of a volumetric sample requires mechanical axial scanning and the acquisition of many successive images (e.g., *N* = 101 here spans ± 500 µm in depth). Bright-field holography, enabled by deep learning, fuses the volumetric imaging capability of holography with the speckle- and artifact-free image contrast of incoherent bright-field microscopy to generate bright-field equivalent images of a volume from a single hologram (*N* = 1 image)

We used a generative adversarial network (GAN)^15–17^ to perform the holographic to bright-field image transformation (Supplementary Fig. S1). The network’s training dataset was made up of images from pollen samples captured on a flat substrate using a sticky coverslip^18^. The coverslip was scanned in 3D using a bright-field microscope (Olympus IX83, 20 × 0.75 NA objective lens), and a stack of 121 images with an axial spacing of 0.5 µm was captured for each region of interest to constitute the ground-truth labels. Next, using a lensless holographic microscope^18^, in-line holograms were acquired corresponding to the same fields of view (FOVs) scanned with the bright-field microscope. By progressively applying a series of image-registration steps from the global coordinates to the local coordinates of each image patch, the backpropagated holograms at different depths were precisely matched to the bright-field microscopy ground-truth image stack in both the lateral and axial directions (see Supplementary Fig. S2 and the Supplementary Information for details). These registered pairs of backpropagated holograms and bright-field microscopy images were then cropped into ~6000 patches of 256 × 256 pixels for training.

It should be emphasized that these steps need to be performed only once for the training of the GAN architecture, after which the generator network can blindly take a new hologram that it has never seen before and infer the corresponding bright-field image at any arbitrary depth within the sample volume in nearly real time (e.g., the inference time for a FOV of ~0.15 mm^2^ is ~0.1 s using a single Nvidia 1080 Ti GPU). Figure 2 presents an example of these blind-testing results for several pollen mixtures, where the backpropagated holograms are compromised by twin-image and self-interference artifacts, as well as speckle and out-of-focus interference. On the other hand, the generator network’s output image for each depth clearly shows improved contrast and is free of the artifacts and noise features observed in the backpropagated holograms. These results match well with the corresponding bright-field images (the ground truth) at the same sample depths.

**Fig. 2:**
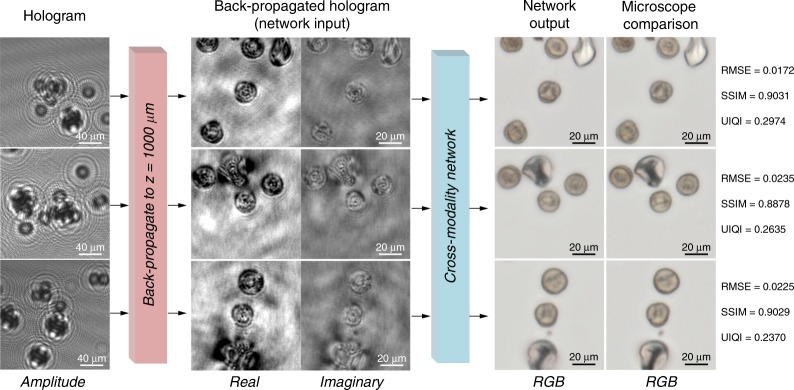
Imaging of a pollen mixture captured on a substrate. Each input hologram is shown with a larger FOV to better illustrate the fringes. Each network output image is quantitatively compared against the corresponding bright-field microscopy ground-truth image using the root mean square error (RMSE), the structural similarity index (SSIM), and the universal image quality index (UIQI)

In addition, the deep network correctly colorizes the output images based on the morphological features in the complex-valued input image, using an input hologram acquired with a monochrome sensor (Sony IMX219PQ, 1.12-µm pixel size) and narrowband illumination (*λ* = 850 nm, bandwidth ~1 nm), such that the output matches the color distribution of the bright-field ground-truth image. This can be seen in Fig. 2 for the yellow ragweed pollens and oak tree pollens, as well as the white Bermuda grass pollens. Furthermore, the root mean square error, structural similarity index^19^, and universal image quality index^20^ were used to quantitatively demonstrate the close resemblance of the network inferences to the bright-field microscopy ground-truth images, as shown in Fig. 2. In addition, we quantitatively compared the performance of several variations of this GAN framework, including one without adversarial loss, one with spectral normalization added to the discriminator, and one with an encoder–decoder structure; the results of these comparisons revealed that these GAN variations demonstrate similar inference performance (see Supplementary Table S1 and Fig. S3 for details).

Although our deep network was trained only with pollen mixtures captured on 2D substrates, it can successfully perform inference for the volumetric imaging of samples at different depths. Figure 3a, b illustrates a pollen mixture captured in 3D in a bulk volume of polydimethylsiloxane (PDMS) with a thickness of ~800 µm. A single in-line hologram of this sample (Fig. 3c) was captured and numerically backpropagated to different depths within the sample volume. By feeding these backpropagated holographic images into our trained network, we obtained output images (Fig. 3) that are free of speckle artifacts and various other interferometric artifacts observed in holography (e.g., twin images, fringes related to out-of-focus objects, and self-interference). These images match the contrast and depth of field (DOF) of bright-field microscopy images that were mechanically focused onto the same plane within the 3D sample (also see Movie 1 for details).

**Fig. 3:**
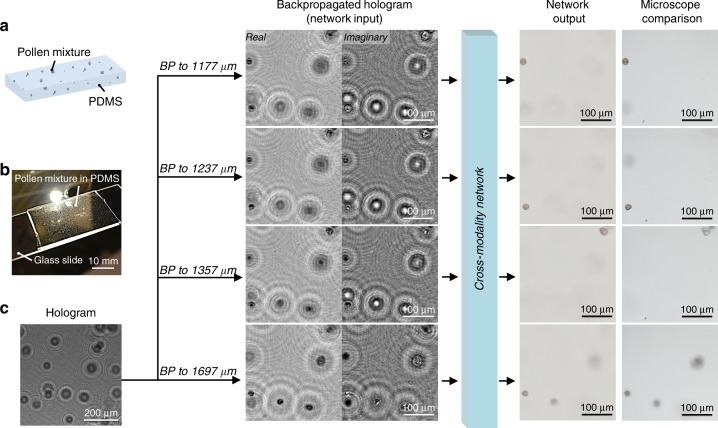
Use of cross-modality deep learning in bright-field holography to fuse the volumetric imaging capability of holography with the speckle- and artifact-free image contrast performance of incoherent bright-field microscopy. The pollen sample is dispersed in 3D throughout a bulk volume of PDMS (thickness ~800 µm). BP: digital backpropagation. Also see Movie 1

For much denser or spatially connected 3D samples, the network’s inference process may generate suboptimal results because our training image data were acquired from uniform and relatively sparse samples (bioaerosols), and in the case of a spatially dense or connected sample, the reference wave in the hologram formation might become distorted because of the in-line operation, deviating from a plane wave due to dense scattering and possible intra-sample occlusion. For applications related to, e.g., aerosol imaging or cytometry, this phenomenon does not pose a limitation; for other applications that require the imaging of denser samples in 3D, the inference performance of our approach can be improved by training the network with dense and spatially connected samples.

It is also worth noting that the snapshot volumetric reconstruction performance presented in this work cannot be obtained through standard coherent denoising or phase-recovery methods. To provide an example of this, in Supplementary Fig. S4, we compare the results of an object-support-based phase-recovery method^7,8^ applied to the same sample hologram that was backpropagated to different heights. As shown in this figure, the iterative phase-recovery method indeed improved the contrast-to-noise ratio (CNR)^21^ of the backpropagated holographic images from ~2 to ~3, especially suppressing some of the twin-image-related artifacts. However, the out-of-focus fringes created by the 3D object were not adequately sectioned out and remained as reconstruction artifacts even after iterative phase recovery. In contrast, the deep neural network output transformed the defocused coherent fringes into diminished incoherent blobs, achieving a high CNR of >15–25, very well matching the ground-truth images captured by the high-NA bright-field microscope, as shown in Supplementary Fig. S4.

To further quantify this cross-modality transformation performance, we imaged samples containing 1-μm polystyrene beads and trained another GAN following the same method (see the Supplementary Information for details). Next, we blindly tested a sample containing 245 individual/isolated microbeads and measured their 3D PSF distributions before and after GAN inference (Fig. 4). An example of this comparison is shown in Fig. 4a, where the backpropagated holograms contain significant interference artifacts that were removed by the GAN, yielding images that match the high contrast of the mechanically scanned bright-field microscopy ground-truth images. Figure 4b shows the distributions of the lateral and axial full-width-at-half-maximum (FWHM) values corresponding to the 3D PSFs obtained using these 245 microbeads. Due to the interference artifacts and low contrast, the FWHM values of the PSFs of the backpropagated hologram (input) are randomly distributed in the lateral direction, with a median FWHM of 2.7176 µm. In contrast, the lateral FWHM values of the PSFs of the GAN output images are monodisperse, with a median FWHM of 1.8254 µm, matching that of the scanning bright-field microscopy ground truth (1.8719 µm). Due to the longer coherence length, the PSFs of the backpropagated hologram (input) are longer in the axial direction, with a median FWHM of 12.9218 µm, compared with the scanning bright-field microscopy ground truth, with a median FWHM of 9.8003 µm. The network inference results show a significantly narrower PSF distribution in the axial direction, with a median FWHM of 9.7978 µm, very well matching that of the ground truth obtained with the scanning bright-field microscope (Fig. 4b). These results and the quantitative agreement between our network output images and the ground-truth images obtained with a scanning bright-field microscope further support the validity of our presented approach.

**Fig. 4:**
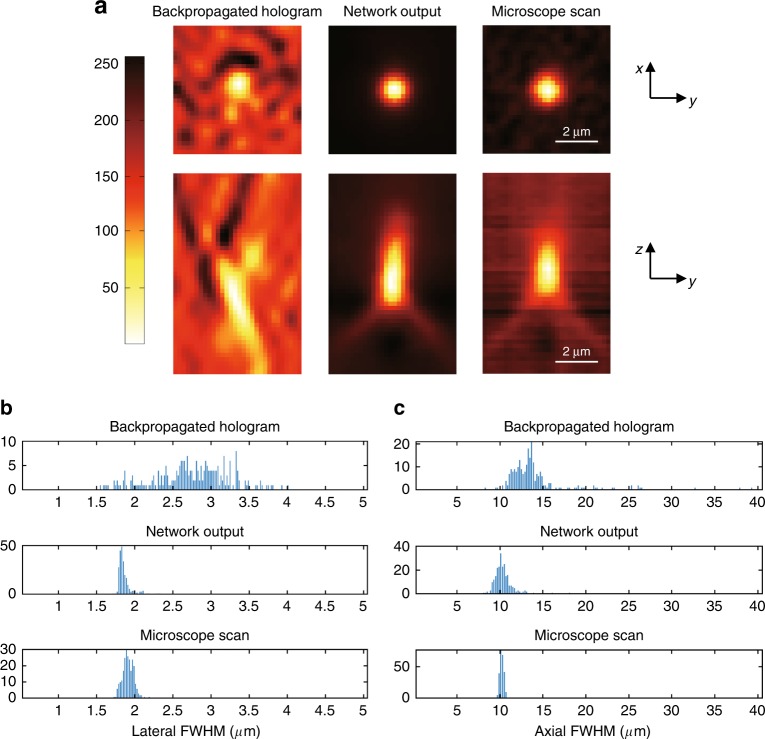
3D PSF comparison using 1-μm beads. **a** 3D imaging of a single microbead and a comparison of the standard holographic backpropagation results against the network output and the images captured by a scanning bright-field microscope via *N* = 81 scans with an axial step size of 0.5 µm. **b** Lateral PSF FWHM histogram comparison corresponding to 245 individual/isolated microbeads. **c** Same as in (**b**), except for the axial PSF FWHM histograms

This deep-learning-enabled cross-modality image transformation between holography and bright-field imaging can potentially eliminate the need to mechanically scan a volumetric sample. It benefits from the digital wave-propagation framework of holography to virtually scan throughout the sample volume, and each one of these digitally propagated fields is transformed into bright-field microscopy equivalent images that exhibit the spatial and color contrast, as well as the shallow DOF expected from incoherent microscopy. In this regard, our deep-learning-enabled hologram transformation network achieves the best of both worlds by fusing the volumetric digital imaging capability of holography with the speckle- and artifact-free image contrast of bright-field microscopy. This capability can be especially useful for the rapid volumetric imaging of samples flowing within a liquid^22^. This approach can also be applied to other holographic microscopy and/or incoherent microscopy modalities to establish a statistical image transformation from one mode of coherent imaging into another incoherent microscopy modality. Further highlighting the importance of data-driven cross-modality image transformations, recent work has demonstrated various other important applications of this general framework, including image superresolution^23^ and label-free virtual staining of pathology samples^24^. Different from these earlier contributions, which transform a 2D sample image into another 2D image, this work enables the inference of a whole 3D sample volume from a single-snapshot hologram, thus reintroducing coherent holographic imaging as a powerful alternative to high-NA bright-field microscopy for the task of high-throughput volumetric imaging, and therefore represents a unique contribution to the field of coherent microscopy.

## Methods

### Digital holographic image acquisition

The holographic images were acquired using a customized lens-free imaging system (see Supplementary Fig. S5 for details). The system consisted of a vertical-cavity surface-emitting laser diode (*λ* = 850 nm) for illumination, a complementary metal–oxide–semiconductor image sensor (Sony IMX219PQ, 1.12-µm pixel size), and a Raspberry Pi 2 for system control. This near-infrared illumination was chosen to enable the use of all four Bayer channels of the color image sensor chip to improve the pixel-size-limited resolution of the hologram that could be achieved in a single snapshot. The sample was mounted on a 3D-printed sample holder placed ~500 µm above the image sensor surface. The illumination source was placed ~8 cm above the sample plane without any additional spatial or spectral filter.

### Scanning bright-field microscopy image acquisition and alignment

The bright-field microscopy images were captured by an inverted scanning microscope (IX83, Olympus Life Science) using a 20 × 0.75 NA objective lens (UPLSAPO20X, Olympus Life Science). The microscope scanned each sample at different lateral locations, and at each location, an image stack of −30 to 30 µm with a 0.5-µm step size was captured. After the capture of these bright-field images, the microscopy image stack was aligned using the ImageJ plugin StackReg^25^, which corrected the rigid shift and rotation caused by the inaccuracy of the microscope scanning stage.

### Hologram backpropagation and autofocusing

The raw digital in-line hologram was balanced and shade corrected by estimating the low-frequency shade of each Bayer channel using a wavelet transform^26^. This corrected hologram was digitally backpropagated to different planes (which matched the corresponding planes in the bright-field microscopy image stack) using angular-spectrum-based free-space backpropagation^4,27^. For this purpose, 3× padding was used in the angular-spectrum (Fourier) domain, which effectively interpolated the hologram pixel size by 3×. To match the heights of the backpropagated holograms and the corresponding bright-field microscopy image stacks, the focal planes were estimated and cross-registered as “zero” height, and the relative axial propagation distance was determined to match the axial scanning step size of the bright-field microscope (0.5 µm). The digital hologram’s focal plane was estimated using an edge sparsity-based holographic autofocusing criterion^28^.

### Network and training

The GAN implemented here consisted of a generator network and a discriminator network, as shown in Supplementary Fig. S1. The generator network employed a variation of the original U-Net^29^ design with minor modifications and additional residual connections^30^. The discriminator network was a convolutional neural network with six convolutional blocks and two fully connected (linear) layers. The original training data consisted of ~6000 image pairs (see Supplementary Table S2 for details), which were augmented to 30,000 image pairs by random rotation and flipping of the images. The validation data were not augmented. In each training iteration, the generator network was updated six times using the adaptive moment estimation (Adam) optimizer with a learning rate of 10^−^^4^, whereas the discriminator network was updated three times with a learning rate of 3 × 10^−5^. The validation set was tested every 50 iterations, and the best network was chosen to be the one with the lowest mean absolute error loss on the validation set. The network was built using an open-source deep-learning package, TensorFlow^31^. The training and inference were performed on a PC with a six-core 3.6-GHz CPU and 16 GB of RAM using an Nvidia GeForce GTX 1080 Ti GPU. On average, the training process took ~90 h for ~50,000 iterations (equivalent to ~40 epochs). After training, the network inference time was ~0.1 s for an image patch of 256 × 256 pixels (see the Supplementary Information for details).

## Supplementary information

Supplementary Information

Movie 1. Bright-field holographic imaging of a 3D pollen stack using our deep learning-based inference and its comparison to a scanning bright-field microscope
