# Supplementary material for: Bright-field holography: cross-modality deep learning enables snapshot 3D imaging with bright-field contrast using a single hologram
Source: Light Sci Appl. 2019 Mar 6;8:25. doi: 10.1038/s41377-019-0139-9 (PMC6401162; doi:10.1038/s41377-019-0139-9)
Supplement: Supplementary file 1 — Supplementary Information [file 41377_2019_139_MOESM1_ESM.pdf]

Supplementary information for:

Bright-field Holography: Cross-modality deep learning enables snapshot 3D imaging with bright-field contrast using a single hologram

Authors: Yichen Wu[1,2,3,†], Yilin Luo[1,2,3,†], Gunvant Chaudhari[4,†], Yair Rivenson[1,2,3,†], Ayfer Calis[1,2,3], Kevin de Haan[1,2,3], Aydogan Ozcan[1,2,3,4,*]

Affiliations:

[1]Electrical and Computer Engineering Department, University of California, Los Angeles, California 90095, USA

[2]Bioengineering Department, University of California, Los Angeles, California 90095, USA

[3]California Nano Systems Institute (CNSI), University of California, Los Angeles, California 90095, USA

[4]David Geffen School of Medicine, University of California, Los Angeles, California 90095, USA

[†]Equally contributing authors

[*]Correspondence: Prof. Aydogan Ozcan

E-mail: ozcan@ucla.edu

Address: 420 Westwood Plaza, Engr. IV 68-119, UCLA, Los Angeles, CA 90095, USA

Tel: +1(310)825-0915

Fax: +1(310)206-4685

Email addresses of all the authors:

Yichen Wu: wuyichen@ucla.edu

Yilin Luo: yilinluo@ucla.edu

Gunvant Chaudhari: gunvantc@ucla.edu

Yair Rivenson: rivenson@gmail.com

Ayfer Calis: ayfercalis@gmail.com

Kevin de Haan: kdehaan@ucla.edu

Aydogan Ozcan: ozcan@ucla.edu

Supplementary Materials and Methods

Sample preparation

Dried pollen samples: Bermuda grass pollen (Cynodon dactylon), oak tree pollen (Quercus agrifolia), and ragweed pollen (Artemisia artemisifolia) were purchased from Stallergenes Greer (NC, USA) (cat #: 2, 195, and 56 respectively) and mixed with a weight ratio of 2 : 3 : 1. The mixture was deposited onto a sticky coverslip from an impaction based air sampler for the 2D pollen sample. The mixture was also diluted into PDMS and cured on a glass slide for the 3D pollen sample.

Polystyrene bead sample with 1 μm diameter was purchased from Thermo Scientific (cat #: 5100A) and diluted 1000× by methanol. A droplet of 2.5 μL of diluted bead sample was pipetted onto a cleaned #1 coverslip and let dry.

Training data preparation

The success of the cross-modality transform behind bright-field holography relies on accurate registration of the back-propagated holograms with the scanning bright-field microscope images in 3D. This registration can be divided into two parts, also shown in Supplementary Fig. S2. The first part matches a bright-field image (2048 × 2048 pixels) to that of the hologram, with the following steps: (1) A stitched bright-field full-FOV image of ~20,000 × 4,000 pixels was generated by stitching together the middle planes of each bright-field microscope stack using ImageJ plugin Microscopy Image Stitching Tool (MIST)[1]. (2) The shade-corrected full-FOV hologram was back-propagated to a global focus distance determined by auto-focusing on a region of 512×512 pixels in the center of the hologram. (3) The bright-field full-FOV image was roughly registered to the back-propagated hologram full-FOV by fitting a rigid transformation through 3-5 pairs of manually selected matching points. (4) The bright-field full-FOV was then warped using this transformation, and the overlapping regions with hologram was cropped to generate matching pairs.

The second part further refines the registration in x-y and z directions, with the following steps: (1) small FOV pairs (300×300 pixels) were selected from the cropped FOV. (2) Autofocusing was performed

on each hologram patch to find the focus distance for this patch, denoted as z_0^{Holo}. (3) The standard deviation (std) of each bright-field height within the stack was calculated, which provides a focus curve for the bright-field stack. A second-order polynomial fit was performed on four heights in the focus curve with highest std values, and the focus for this bright-field stack was determined to be the peak location of the fit, denoted as z_0^{BF}. (4) For each microscope scan in the stack at height z_i^{BF}, a corresponding hologram image was generated by back-propagating the hologram by the distance $z_i^{BF} - z_0^{BF} + z_0^{Holo}$, where symmetric padding was used on the hologram during the propagation to avoid ringing artifacts. (5) The best focused plane in each stack, as well as five other randomly selected defocused planes were chosen. (6) Pyramid elastic registration[2] was performed on the small FOV image pair closest to the focal plane, and the same registered warping was applied to the other five defocused image pairs to generate 6 aligned small FOV pairs in total. (7) The corresponding patches were cropped to 256×256 pixels in image size. Since the pyramidal registration can sometimes fail to converge to the correct transformation, the generated dataset was also manually inspected to remove the data that had significant artifacts due to registration errors.

Details of network and training

The GAN implemented here consisted of a generator network and a discriminator network, as shown in Supplementary Fig. S1. The generator employed an alternation of the original U-Net[3] design with minor modifications and additional residual connections[4]. The discriminator network was a convolutional neural network with six convolutional blocks and two fully-connected (linear) layers. The input of the generator had dimensions of 256×256×2 where the two channels were the imaginary and real parts of the back-propagated complex-valued hologram. The output of generator and the input of discriminator had dimensions of 256×256×3, with the three channels corresponding to the red, green and blue (RGB) channels of the bright-field image. Following the image registration and cropping, the dataset was divided into 75% for training, 15% for validation, and 10% for blind testing. The training data consisted of ~6,000

image pairs, which were further augmented to 30,000 by random rotation and flipping of the images. The validation data were not augmented.

During the training phase, the network iteratively minimized the generator loss L_G and discriminator loss L_D, defined as:

$$L_G = \frac{1}{2N} \cdot \sum_{i=1}^{N} \left[D\left(G\left(x^{(i)}\right) \right) - 1 \right]^2 + \alpha \cdot \frac{1}{N} \cdot \sum_{i}^{N} MAE\left(x^{(i)}, z^{(i)}\right) \quad (1)$$

$$L_D = \frac{1}{2N} \cdot \sum_{i=1}^{N} \left[D\left(G\left(x^{(i)}\right) \right) \right]^2 + \frac{1}{2N} \cdot \sum_{i=1}^{N} \left[D\left(z^{(i)}\right) - 1 \right]^2 \quad (2)$$

where $G\left(x^{(i)}\right)$ is the generator output for the input $x^{(i)}$, $z^{(i)}$ is the corresponding target (bright-field) image, $D(.)$ is the discriminator, and $MAE(.)$ stands for the mean absolute error, defined as:

$$MAE(x, z) = \frac{1}{L^2} \sum_{m=1}^{L} \sum_{n=1}^{L} \left| x_{mn} - z_{mn} \right| \quad (3)$$

where the images have L×L pixels. N stands for the image batch size (e.g., N = 20), α is a balancing parameter for the GAN loss and the MAE loss in the L_G which was chosen as α =0.01 and as result, the GAN loss and MAE loss terms occupied 99% and 1% of the total loss, L_G, respectively. Adaptive momentum (Adam) optimizer was used to minimize L_G and L_D, with learning rate 10^{-4} and 3×10^{-5} respectively. In each iteration, six updates of the generator and three updates of the discriminator network were performed. The validation set was tested every 50 iterations, and the best network was chosen to be the one with the lowest MAE loss on the *validation* set. The network was implemented using TensorFlow[5].

Estimation of the lateral and axial FWHM values for PSF analysis

A threshold was used on the most focused hologram plane to extract individual sub-regions, each of which contained a single bead. A 2D Gaussian fit[6] was performed on each sub-region to estimate the lateral PSF FWHM. The fitted centroid was used to crop x-z slices, and another 2D Gaussian fit was

performed on each slice to estimate the axial PSF FWHM values for (i) the back-propagated hologram stacks, (ii) the network output stacks and (iii) the scanning bright-field microscope stacks. Histograms for the lateral and axial PSF FWHM were generated subsequently, as shown in Fig. 4.

Quantitative evaluation of image quality

Each network output image I^{out} was evaluated with reference to the corresponding ground truth (bright-field microscopy) image I^{GT} using four different criteria: (1) root mean square error (RMSE), (2) correlation coefficient (Corr), (3) structural similarity (SSIM) [7], and (4) universal image quality index (UIQI) [8]. RMSE is defined as:

$$\mathrm{RMSE}\left(I^{out}, I^{GT}\right) = \frac{1}{\sqrt{L_x \cdot L_y}} \sqrt{\| I^{out} - I^{GT} \|_2^2} \quad (4)$$

where L_x and L_y represent the number of pixels in the x and y directions, respectively.

Correlation coefficient is defined as:

$$\mathrm{Corr}\left(I^{out}, I^{GT}\right) = \frac{\sigma_{out,GT}}{\sigma_{out}\sigma_{GT}} \quad (5)$$

where σ_{out} *and* σ_{GT} are the standard deviations of I^{out} *and* I^{GT} respectively, and $\sigma_{out,GT}$ is the cross-variance between the two images.

SSIM is defined as:

$$\mathrm{SSIM}\left(I^{out}, I^{GT}\right) = \frac{\left(2\mu_{out}\mu_{GT} + C_1\right)\left(2\sigma_{out,GT} + C_2\right)}{\left(\mu_{out}^2 + \mu_{GT}^2 + C_1\right)\left(\sigma_{out}^2 + \sigma_{GT}^2 + C_2\right)} \quad (6)$$

where μ_{out} *and* μ_{GT} are the mean values of the images I^{out} *and* I^{GT}, respectively. C_1 and C_2 are constants used to prevent division by a denominator close to zero.

UIQI is the product of three components: correlation coefficient (Corr, see Eq. (5)), luminance distortion (l) and contrast distortion (c), i.e.:

$$\mathrm{UIQI}(\mathrm{I^{out}}, \mathrm{I^{GT}}) = \mathrm{Corr}(\mathrm{I^{out}}, \mathrm{I^{GT}}) \bullet \mathrm{l}(\mathrm{I^{out}}, \mathrm{I^{GT}}) \bullet \mathrm{c}(\mathrm{I^{out}}, \mathrm{I^{GT}}) \quad (7)$$

where

$$\mathrm{l}(\mathrm{I^{out}}, \mathrm{I^{GT}}) = \frac{2\mu_{out}\mu_{GT}}{\mu_{out}^2 + \mu_{GT}^2} \quad (8)$$

$$\mathrm{c}(\mathrm{I^{out}}, \mathrm{I^{GT}}) = \frac{2\sigma_{out}\sigma_{GT}}{\sigma_{out}^2 + \sigma_{GT}^2} \quad (9)$$

UIQI was measured locally across *M* windows of size $B \times B$, generating *local* UIQIs: Q_i $(i = 1,2, \dots, M)$. Then the *global* UIQI was defined as the average of these local UIQIs:

$$Q = \frac{1}{M}\sum_{i=1}^{M} Q_i \quad (10)$$

We used a window of size *B* = 8, same as in Ref. 8.

In addition to the above discussed measures, we also evaluated the image quality using the Blind Reference-less Image Spatial Quality Evaluator (BRISQUE), using a Matlab built-in function "brisque"[9].

References

1. Chalfoun, J. *et al.* MIST: Accurate and Scalable Microscopy Image Stitching Tool with Stage Modeling and Error Minimization. *Sci. Rep.* **7**, 4988 (2017).
2. Rivenson, Y. *et al.* Deep Learning Enhanced Mobile-Phone Microscopy. *ACS Photonics* **5**, 2354–2364 (2018).
3. Ronneberger, O., Fischer, P. & Brox, T. U-Net: Convolutional Networks for Biomedical Image Segmentation. *ArXiv150504597 Cs* (2015).
4. He, K., Zhang, X., Ren, S. & Sun, J. Deep Residual Learning for Image Recognition. in 770–778 (2016).

5. Abadi, M. *et al.* TensorFlow: A System for Large-Scale Machine Learning. in *OSDI* **16**, 265–283 (2016).

6. Fit 2D gaussian function to data - File Exchange - MATLAB Central. Available at: https://www.mathworks.com/matlabcentral/fileexchange/37087. (Accessed: 9th November 2018)

7. Wang, Z., Bovik, A. C., Sheikh, H. R. & Simoncelli, E. P. Image quality assessment: from error visibility to structural similarity. *IEEE Trans. Image Process.* **13**, 600–612 (2004).

8. Wang, Z. & Bovik, A. C. A universal image quality index. *IEEE Signal Process. Lett.* **9**, 81–84 (2002).

9. Blind/Referenceless Image Spatial Quality Evaluator (BRISQUE) no-reference image quality score - MATLAB brisque. Available at: https://www.mathworks.com/help/images/ref/brisque.html#d120e8865. (Accessed: 3rd February 2019)

10. Mudanyali, O. *et al.* Compact, light-weight and cost-effective microscope based on lensless incoherent holography for telemedicine applications. *Lab. Chip* **10**, 1417–1428 (2010).

11. Fienup, J. Phase Retrieval Algorithms - a Comparison. *Appl. Opt.* **21**, 2758–2769 (1982).

12. Zhang, Y. *et al.* 3D imaging of optically cleared tissue using a simplified CLARITY method and on-chip microscopy. *Sci. Adv.* **3**, e1700553 (2017).

Supplementary Figures and Captions

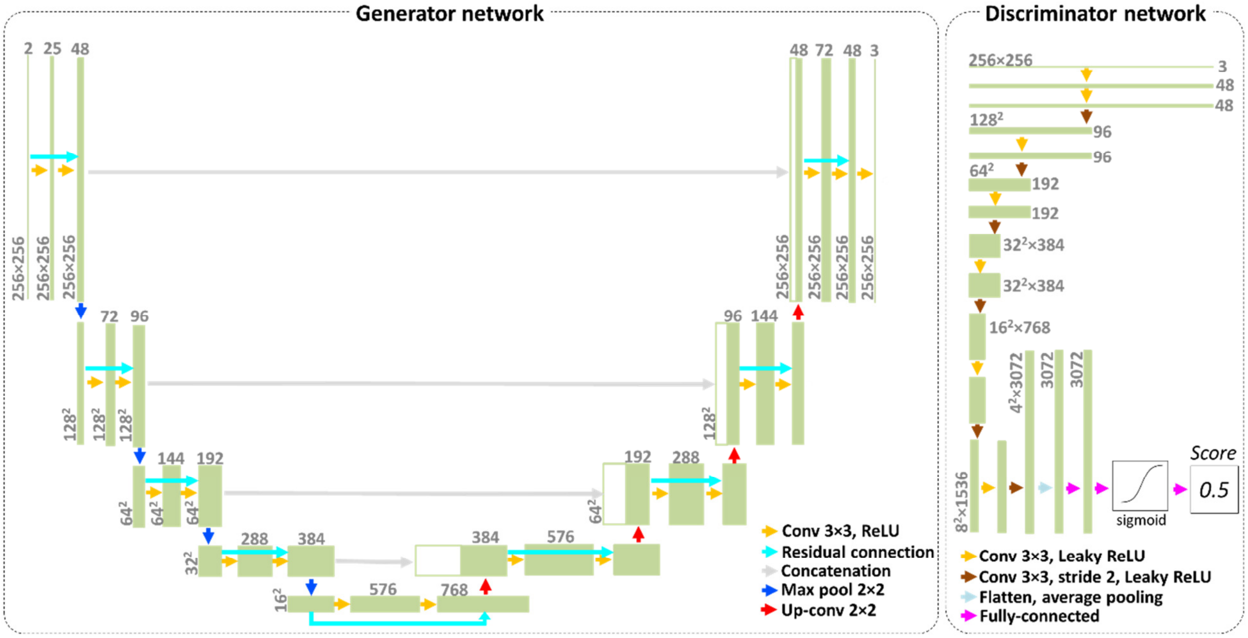

Supplementary Fig. S1. Network structure. The numbers represent the size and the channels of each block. ReLU: rectified linear unit. Conv: convolutional layer.

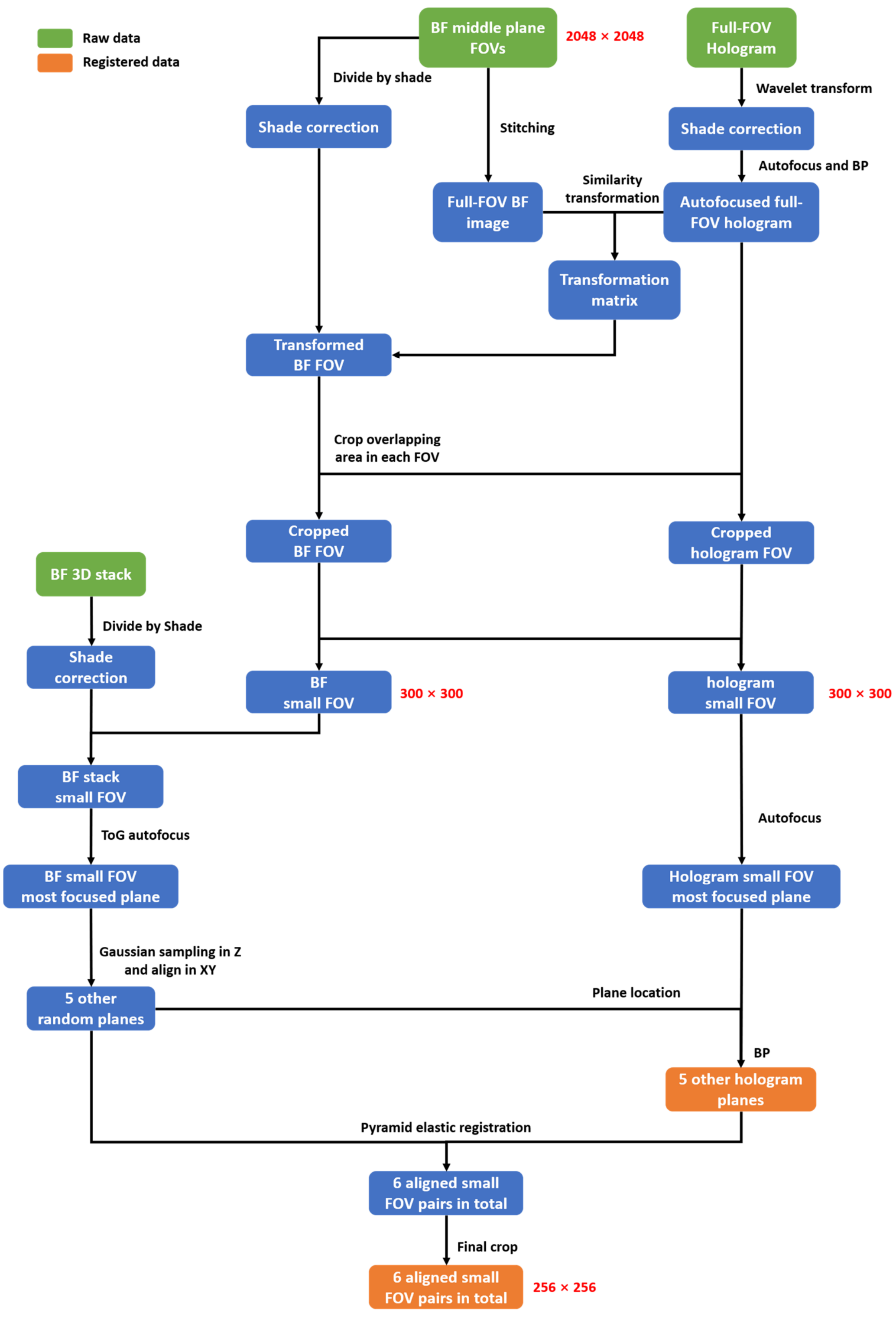

Supplementary Fig. S2. Image registration and data pre-processing flow-chart.

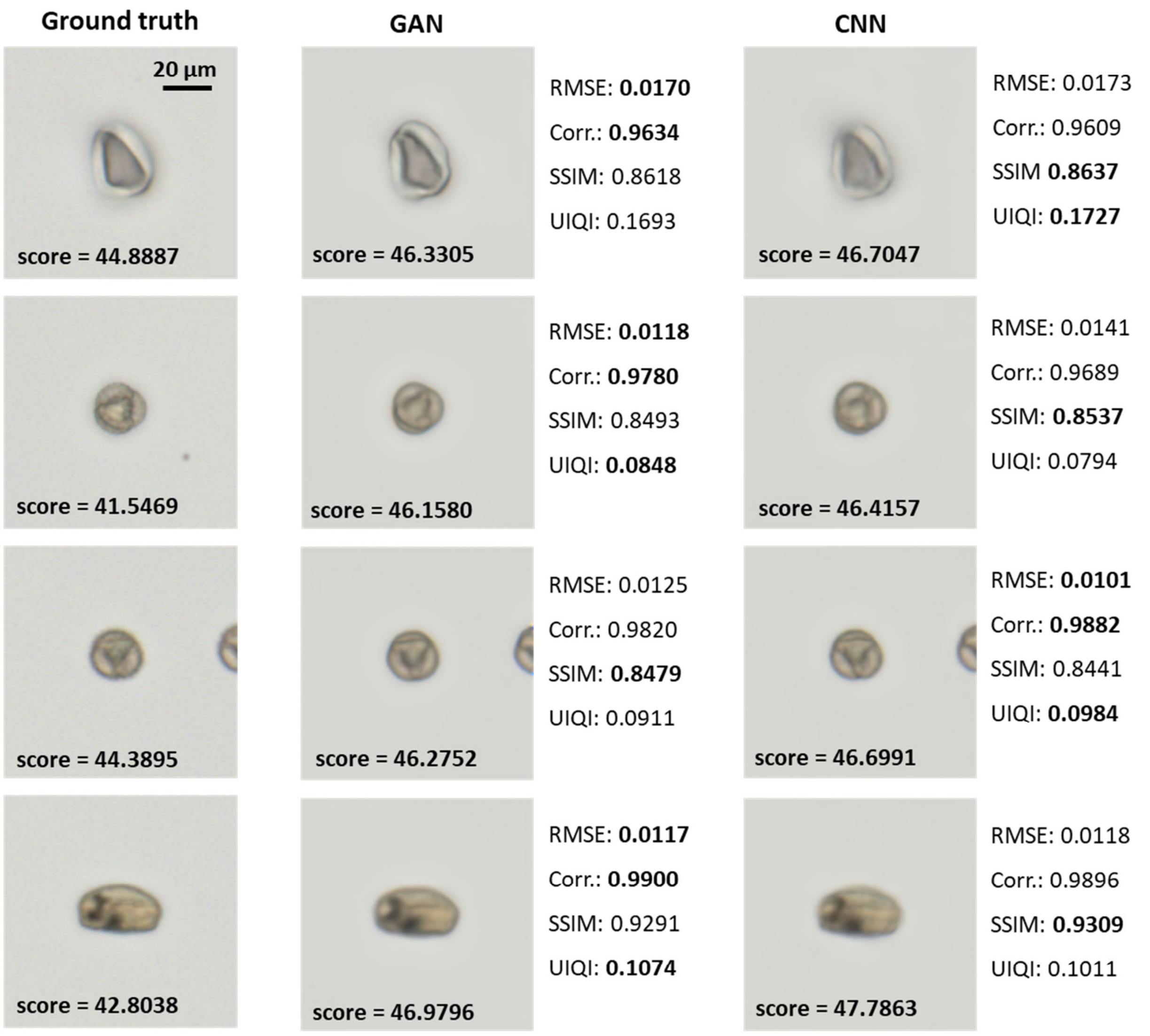

Supplementary Fig. S3. **Sample reconstructions by GAN and CNN.** The GAN framework is the same as the one that is used to report the results in the main text, also detailed in Supplementary Fig. S1. The CNN is the same as the generator network trained *without* using the discriminator and the adversarial loss terms. The two networks were trained on the same dataset for similar number of iterations (~40 epochs). Four in-focus reconstructions of pollen samples are shown. The images are compared against the corresponding microscope image ground truth acquired at the same depth, using root mean square error (RMSE), Pearson correlation coefficient (Corr.), structural similarity index (SSIM), and universal image quality index (UIQI). The better value for each criterion for each image is highlighted in bold. The quantitative values resulting from this comparison are almost identical for GAN and CNN output images.

However, the GAN outputs are sharper and exhibit more information, which are visually more appealing than the CNN outputs. Each sample image is also evaluated using a non-reference Blind/Referenceless Image Spatial Quality Evaluator (BRISQUE) score, where the lower score represents better visual quality. The BRISQUE scores are shown on the lower corner of the images, where GAN output images have a smaller/better score compared to CNN output images.

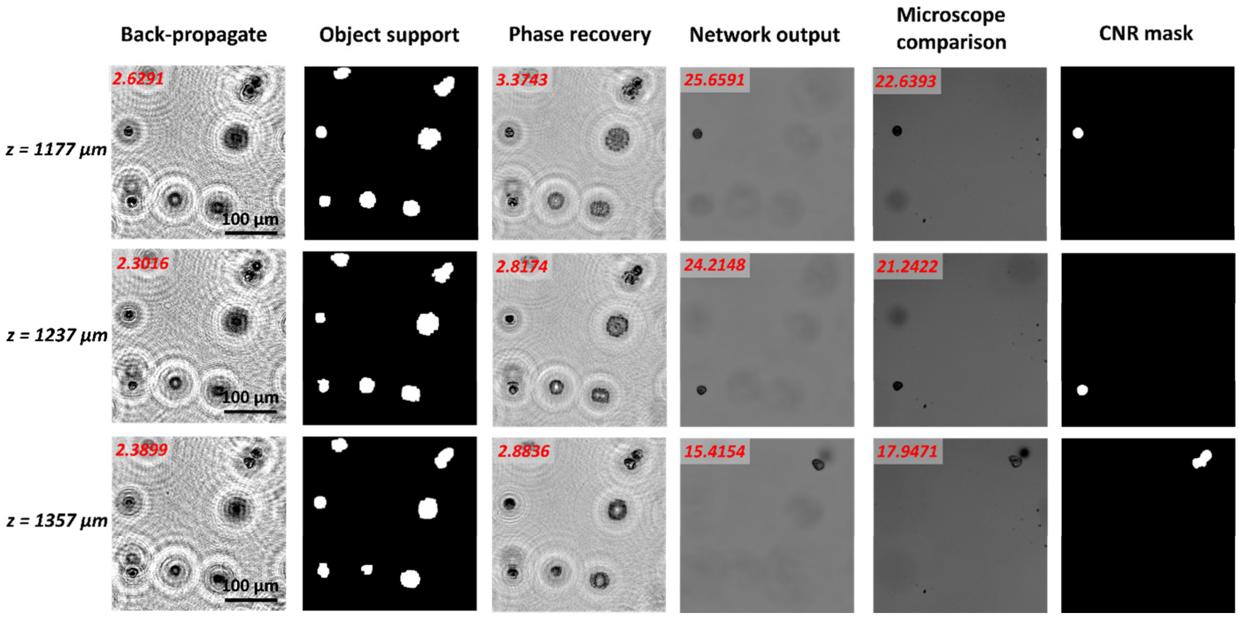

Supplementary Fig. S4. Comparison of bright-field holography against an iterative phase recovery method for a volumetric pollen sample. Object support-based phase recovery[10,11] was used (with 20 iterations). The network output and bright-field microscope ground truth images were converted into gray scale using the Matlab function rgb2gray for comparison purposes. Contrast to noise ratio (CNR)[12], which is defined as the ratio of the contrast (the average of the pixel amplitudes outside the CNR mask minus the average of the pixel amplitudes inside the mask) with respect to noise (the standard deviation of the pixel amplitudes outside the mask), was used to quantitatively compare the results, with the corresponding CNR values marked by a red number on the upper-left corner of each image.

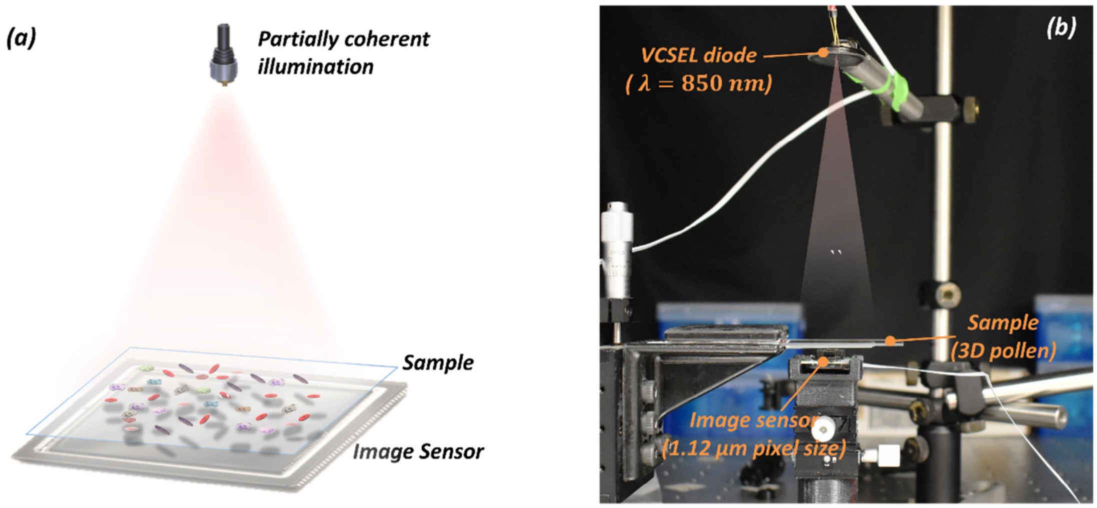

Supplementary Fig. S5. Digital holographic imaging setup. (a) Optical schematic and (b) photo of the setup.

	GAN	CNN	Encoder-decoder	GAN with spectral normalization
RMSE	0.0201	0.0191	0.0218	0.0206
Corr.	0.9643	0.9666	0.9586	0.9626
SSIM	0.8598	0.8567	0.8519	0.8554
UIQI	0.1419	0.1466	0.1345	0.1391

Supplementary Table S1. Quantitative comparison of four different network variations. The GAN is the one that is used to report the results in the main text, which is detailed Supplementary Fig. S1. The CNN is the same generator network without using the discriminator and the adversarial loss. The encoder-decoder structure was constructed by removing the concatenation connections in the U-Net (gray arrows in Supplementary Fig. S1). The GAN with spectral normalization is the same structure as GAN, with spectral normalization performed on each convolutional layer of the discriminator. All the networks were trained on the same dataset for similar number of iterations (~40 epochs). The trained networks were tested on 175 digitally focused, back-propagated holograms of pollen images. The network outputs were quantitatively compared against the corresponding microscope image ground truth acquired at the same depth, using root mean square error (RMSE), Pearson correlation coefficient (Corr.), structural similarity index (SSIM), and universal image quality index (UIQI). The quantitative values resulting from this comparison are similar regardless of these different network variations that were introduced.

Dataset	Training images	Validation images	Testing images	Data distribution
2D pollen dataset	5,966	1,000	175	Range [-30, 30] µm, step 0.5 µm<br>In-focus : defocus = 1 : 5
3D pollen dataset	0	0	1	Range [-500, 500] µm, step 10 µm
1 µm bead dataset	13,603	2,400	245	Range [-20, 20] µm, step 0.5 µm<br>In-focus : defocus = 1 : 21

Supplementary Table S2. Size and distribution of the training and testing data set. The 2D pollen dataset is composed of images from pollen samples captured on a flat substrate using a sticky coverslip. The 3D pollen dataset is composed of images of pollen mixture spread in 3D inside a polydimethylsiloxane (PDMS) substrate with ~ 800 µm thickness. The 3D pollen dataset only has testing images and is evaluated using the network trained with 2D pollen images. Both datasets include in-focus and de-focused pairs of images for training to capture the 3D light propagation behavior across the holographic and bright-field microscopy modalities. The image size of 3D pollen PDMS testing dataset is 1024×1024 pixels, the other images are of size 256×256 pixels.

Caption of Movie 1

Movie 1. Bright-field holographic imaging of a 3D pollen stack using our deep learning-based inference and its comparison to a scanning bright-field microscope. There is a small, uncontrollable, tilt between the measurement of the hologram and the corresponding bright-field microscope stack. To provide better visualization and comparison, a 2D image registration for the network inference and the microscope target at each height in the video was performed. The smaller particles in the target images that do not appear in the network inference are dust particles on the brightfield microscope image sensor chip, which clearly do not defocus as the video focuses to different depths within the 3D sample volume. Therefore, these dust particles do not exist in the holographic imaging set-up or its inference as they only belong to the brightfield microscopy set-up (image sensor).
